# A Novel Antithrombotic Mechanism Mediated by the Receptors of the Kallikrein/Kinin and Renin–Angiotensin Systems

**DOI:** 10.3389/fmed.2016.00061

**Published:** 2016-11-28

**Authors:** Alvin H. Schmaier

**Affiliations:** ^1^University Hospitals Cleveland Medical Center, Case Western Reserve University, Cleveland, OH, USA

**Keywords:** prekallikrein, bradykinin B2 receptor, Mas receptor, angiotensin receptor 2, factor XII, high molecular weight kininogen, prostacyclin, tissue factor and coagulation

## Abstract

The contact activation (CAS) and kallikrein/kinin (KKS) systems regulate thrombosis risk in two ways. First, the CAS influences contact activation-induced factor XI activation and thrombin formation through the hemostatic cascade. Second, prekallikrein (PK) and bradykinin of the KKS regulate expression of three vessel wall G-protein-coupled receptors, the bradykinin B2 receptor (B2R), angiotensin receptor 2, and Mas to influence prostacyclin formation. The degree of intravascular prostacyclin formation inversely regulates intravascular thrombosis risk. A 1.5- to 2-fold increase in prostacyclin, as seen in PK deficiency, increases vessel wall Sirt1 and KLF4 to downregulate vessel wall tissue factor which alone is sufficient to lengthen induced thrombosis times. A twofold to threefold increase in prostacyclin, as seen the B2R-deficient mouse, delays thrombosis and produces a selective platelet function defect of reduced GPVI activation and platelet spreading. Regulation of CAS and KKS protein expression has a profound influence on thrombosis-generating mechanisms in the intravascular compartment.

The contact activation system (CAS) is known to initiate thrombus formation in surface-activated blood coagulation of plasma by factor XII (FXII) autoactivation with subsequent activation of plasma prekallikrein (PK), amplification of FXII and PK activation by each other, and subsequent activation of factor XI initiating a cascade of proteolytic reactions leading to thrombin formation (Figure 1). Plasma high-molecular weight kininogen (HK) accelerates these reactions. In a rabbit model of extracorporeal membrane oxygenation (ECMO), a novel Fab 3F7 directed to FXIIa and betaFXIIa reduced fibrin formation on the membrane oxygenator to the same extent as heparin (1). These data indicate that activations of the CAS leading to thrombin formation are blocked by inhibitors to FXIIa. Also in mice, CAS inhibition results in improved survival of *F12*^−/−^ mice in the surface-activated collagen-, epinephrine-, or long-chain polyP-induced pulmonary embolism models when compared to wild type (WT) (2–4). In humans, FXIIa inhibition is useful to prevent *in vivo* contact activation from sepsis from any cause, adult respiratory distress syndrome, and when human blood comes in contact with artificial surfaces of medical devices such as ECMO, cardiopulmonary bypass, left-ventricular assist devices, and indwelling intravenous catheters (5). HK-deficient mice (*Kgn1*^−/−^) are also protected from thrombosis, but the mechanism for thrombosis delay is less well characterized (6). They appear to be like *F12*^−/−^ mice, since *Kgn1*^−/−^ mice also have improved survival after long-chain polyP infusions (4).

**Figure 1:**
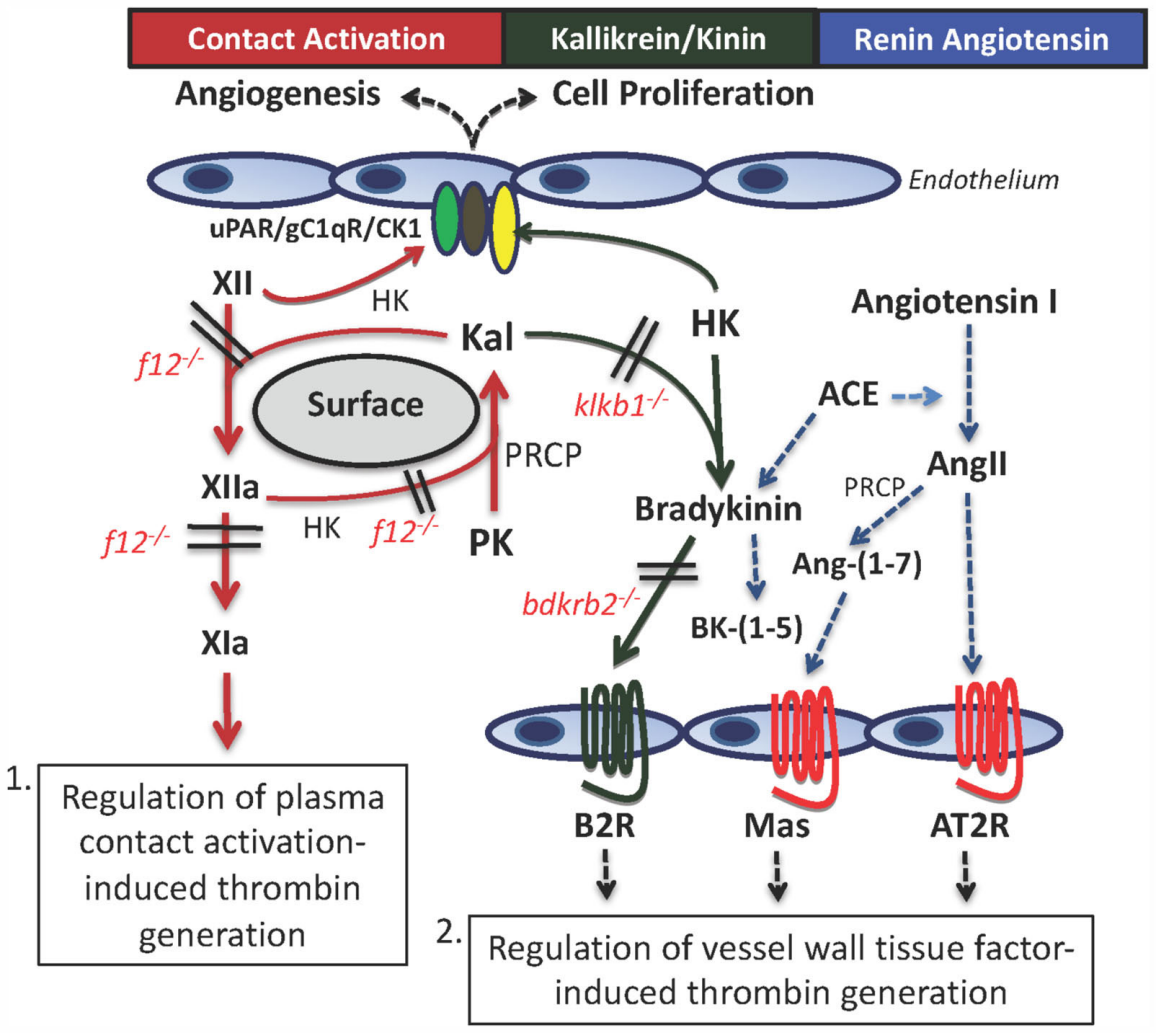
**The contact activation and kallikrein/kinin systems (KKS) have two mechanisms, whereby they influence thrombosis risk in the intravascular compartment**. First, through contact autoactivation of factor XII (XII) to factor XIIa (XIIa), plasma prekallikrein (PK) is activated to plasma kallikrein (Kal) that then reciprocally activates XII and amplifies each other’s activation. Formed XIIa activates factor XI to factor XIa (XIa) that leads to thrombin formation. This pathway occurs in sepsis, adult respiratory distress syndrome, and on mechanical surfaces of devices used in the medical practice. The second pathway for thrombosis risk reduction is through the interaction of the plasma KKS with the renin–angiotensin system (RAS). Formed Kal cleaves bradykinin (BK) from high-molecular weight kininogen (HK), and it binds to the constitutive bradykinin B2 receptor (B2R). It stimulates cells to produce prostacyclin and NO. BK is metabolized by angiotensin-converting enzyme (ACE) that also converts angiotensin I to angiotensin II (AngII). If the angiotensin receptor 2 (AT2R) has more copies than the angiotensin receptor I (not shown), it binds to stimulate prostacyclin and NO. Also, AngII is metabolized to angiotensin-(1–7) [Ang-(1–7)] to stimulate its receptor, Mas, and also to produce prostacyclin and NO. Through the production of prostacyclin, each of these receptors has potential to regulate vessel wall tissue factor and change thrombosis risk within the intravascular compartment.

However, other gene-deleted animals of the plasma CAS and kallikrein/kinin system (KAS) do not behave like *F12*^−/−^ mice on contact activation-induced thrombosis assay. Plasma PK (*Klkb1*^−/−^) and bradykinin B2 receptor knockout (*Bdkrb2*^−/−^) mice do not have increased survival in the collagen-, epinephrine-, or long-chain polyp-induced pulmonary embolism models (5, 7). *Klkb1*^−/−^ mice have reduced contact activation as indicated by delayed contact activation thrombin generation times, long aPTT, and reduced lung edema after collagen–epinephrine insult (7). However, this reduced contact activation is not enough to provide thrombosis protection. Another mechanism is involved in its thrombosis protection. This assessment is the basis of the present review. Deficiencies of proteins of the plasma KKS (PK, bradykinin B2 receptor) allow for a novel mechanism for thrombosis protection through regulation of vessel wall tissue factor (TF) (Figure 1). Characterization of this mechanism is the basis of this report. In this pathway, regulation of vessel wall TF is mediated by vascular G-protein-coupled receptors (GPCRs) of the KKS and renin–angiotensin system (RAS) and their influence on prostacyclin.

## Characterization of *Klkb1*^−/−^ Mice

Detailed mechanistic investigations on PK-deficient mice reveal a previously unappreciated thrombosis protection pathway that modulates *in vivo* thrombosis risk through regulation of vessel wall TF expression. The mechanism for this pathway is not obvious but was discovered by following the data from research observations. When we realized that *Klkb1*^−/−^ mice have expected reduced contact activation, but do not have protection on contact activation-induced thrombosis assays, other mechanisms were sought. We observed that *Klkb1*^−/−^ mice have reduced bradykinin (BK) to about 50% of the level of normal and higher than *Kgn1*^−/−^ mice that have none (6, 7). We had expected that the bradykinin B2 receptor (B2R) that is constitutively expressed would be increased in response to low BK. To our surprise, the B2R was decreased. The B2R is known to make heterodimeric complexes with the angiotensin receptor 2 (AT2R) and the Mas receptor (Mas) (8, 9). Furthermore, the B2R, AT2R, and Mas, when stimulated, all produce prostacyclin and NO. Finally, we knew from previous studies that in the absence of the B2R, AT2R and Mas become overexpressed (see below) (10, 11).

**Figure 2:**
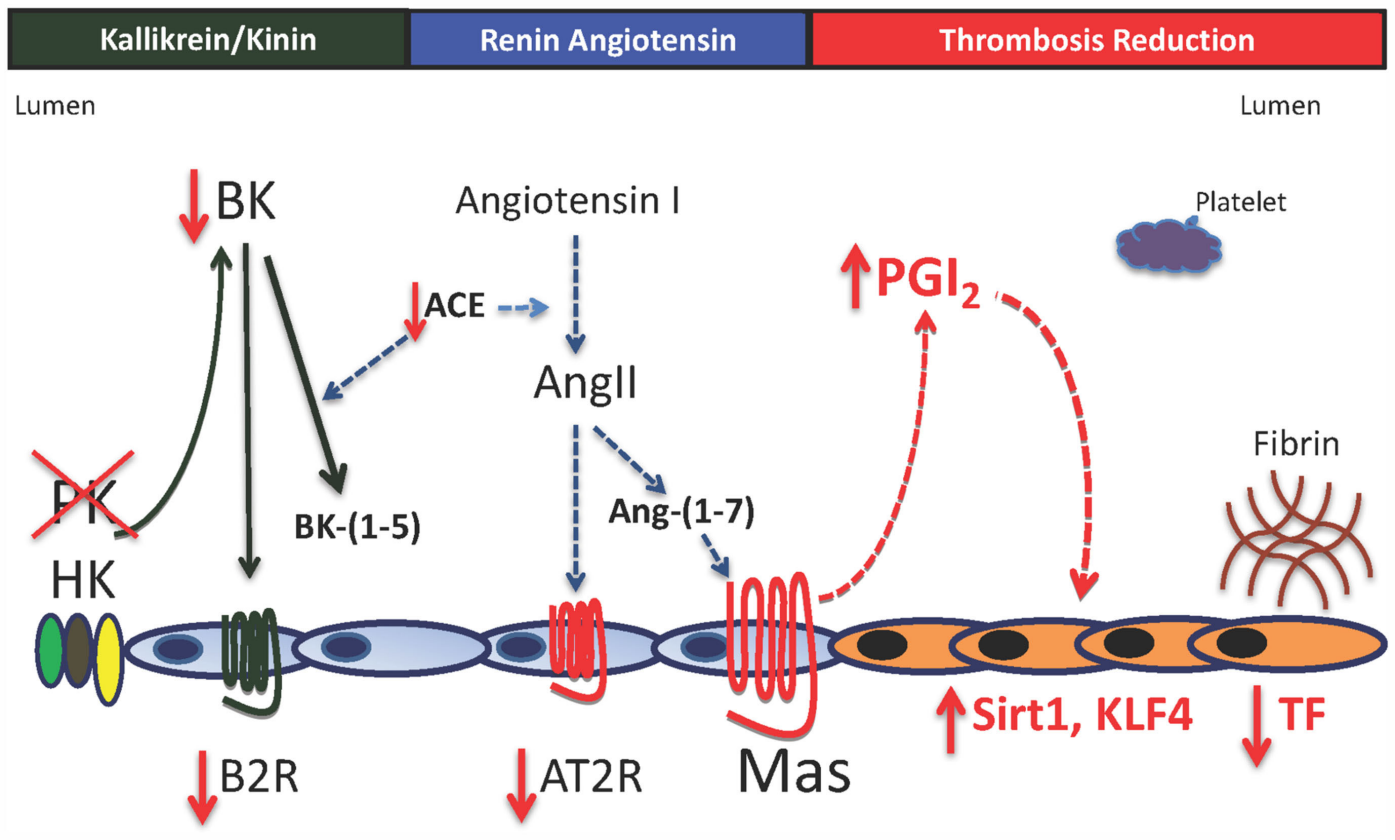
**Model for vessel wall tissue factor regulation in prekallikrein deficiency**. In the absence of plasma prekallikrein (PK), there is reduced bradykinin (BK) and the bradykinin B2 receptor (B2R). This result is also associated with reduced angiotensin-converting enzyme activity (ACE) and the angiotensin receptor 2 (AT2R) with elevation of the G-protein-coupled receptor Mas with normal angiotensin-(1–7) [Ang-(1–7), its natural ligand]. Increased Mas receptor alone is associated with a 1.5- to 2-fold elevation of prostacyclin (PGI_2_) with increased vessel wall Sirt1 and KLF4 and reduced vessel wall tissue factor (TF). There is no platelet function defect in prekallikrein-deficient platelets. The delayed thrombosis in PK-deficient mice is due to reduced vessel wall TF alone.

Investigations next determined if the AT2R and/or Mas receptor levels are increased. We observe increased Mas receptor mRNA and protein with reduced AT2R and angiotensin-converting enzyme (ACE) mRNA and normal angiotensin-(1–7) [Ang-(1–7)] levels (Figure 2). We next determined that a Mas receptor antagonist, A-779, corrects the prolonged thrombosis time in *Klkb1*^−/−^ mice (7). Studies subsequently document that plasma prostacyclin, as measured by its stable plasma breakdown product 6-keto-PGF1α, is elevated, and the Mas antagonist A-779 corrects it to normal and nimesulide, a cyclooxygenase 2 inhibitor, shortens *Klkb1^−/−^* mice thrombosis times to normal (8). Even though *Klkb1*^−/−^ mice have elevated prostacyclin, thrombosis delay is not due to decreased platelet function. *Klkb1*^−/−^ mice have normal bleeding times and platelet function as determined by thrombin-, CRP-, or ADP-induced platelet activation. *Klkb1*^−/−^ mice also have normal platelet spreading on a collagen or fibrinogen matrix. Therefore, other mechanisms were sought.

*Klkb1*^−/−^ mice have reduced platelet adherence on the cremasteric mouse laser injury thrombosis model. Since they have normal platelet function, we asked if there were reduced vessel wall TF (8). Aortic and carotid artery TF mRNA, protein, activity, and expression were reduced. Furthermore, plasma of *Klkb1*^−/−^ mice has normal TF-induced thrombin generation times when 5 pM rTF is used. However, when 0.5 pM TF is employed, plasma from *Klkb1*^−/−^ mice has reduced TF-induced thrombin generation. Recent studies by Barbieri and coworkers show that COX2^−/−^ mice have increased vessel wall TF mediated by reduced prostacyclin, allowing for reduction of the vasculoprotective transcription factor Sirt1 (12). We determined if *Klkb1*^−/−^ mice have increased sirtuin 1 (silent mating type information regulation 2 homolog 1) (Sirt1) and Kruppel-like factor 4 (KLF4), another vasculoprotective transcription factor. *Klkb1*^−/−^ mice have increased Sirt1 and KLF4 mRNA and protein and on immunofluorescence, increased antigen is observed in the smooth muscle layer and endothelium, respectively (7). Since Sirt1 is a histone deacetylase that binds p65 of NFκB and KLF4 binds the p300 co-activator of NFκB, the lowering of vessel wall TF in *Klkb1*^−/−^ mice is not surprising. What is surprising in these animals is the recognition that modulation of vessel wall TF alone in *Klkb1*^−/−^ mice is sufficient to alter *in vivo* thrombosis risk in mice. This finding was unexpected and indicates that the Mas–prostacyclin axis is a mechanism for thrombosis risk regulation. A summary of this mechanism for thrombosis protection in *Klkb1*^−/−^ mice is shown in Figure 2.

## Characterization of *Bdkrb2*^−/−^ Mice

**Figure 3:**
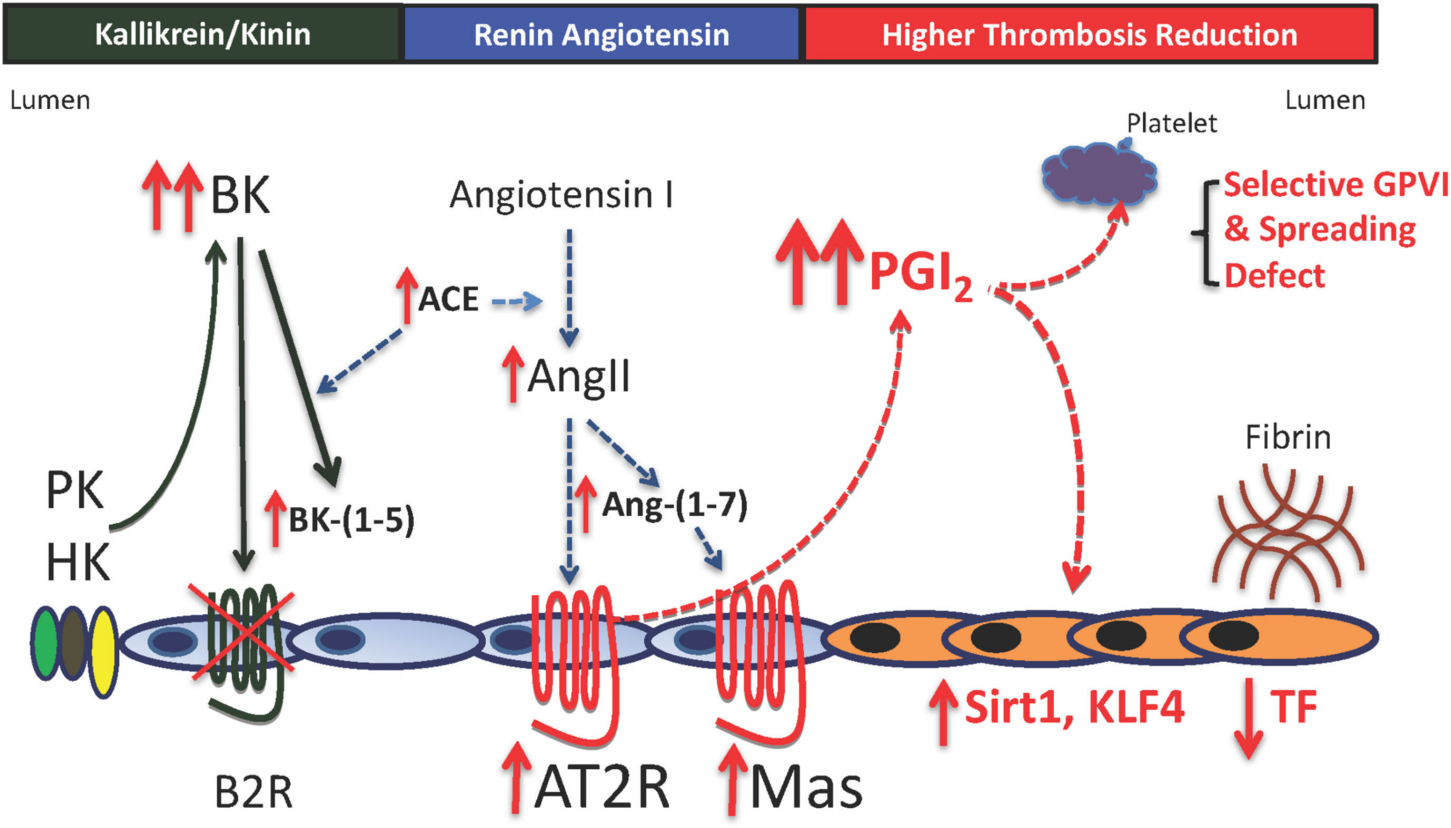
**Mechanisms for thrombosis delay in the bradykinin B2 receptor-deleted mice**. In the absence of the bradykinin B2 receptor (B2R), there is increased plasma bradykinin (BK), its ACE breakdown product, BK-(1–5) (RPPGF). There is also increased ACE, angiotensin II (AngII), and angiotensin-(1–7) [Ang-(1–7)]. Elevated AngII and Ang-(1–7) bind to increased angiotensin receptor 2 (AT2R) and Mas to increase prostacyclin (PGI_2_) levels twofold to threefold above normal. This elevation in plasma PGI_2_ results in increased vessel wall Sirt1 and KLF4 with reduced TF and a selective platelet function defect. The selective platelet function defect is reduced GPVI activation with normal thrombin- and ADP-induced platelet activation and reduced spreading on collagen and adhesive glycoproteins through β1 integrin binding. The delayed thrombosis in B2R-deficient mice is reduced vessel wall tissue factor a selective platelet function defect of GPVI activation and spreading.

We next asked if the thromboprotection mechanism seen in *Klkb1*^−/−^ mice is observed in any other animals? Previous investigations in our laboratory show that *Bdkrb2*^−/−^ mice also have delayed times to arterial thrombosis (10, 11). Our investigations reveal that in the absence of the B2R, both the AT2R and Mas receptors are increased as determined by mRNA and protein in kidney and aorta of these animals. Unlike *Klkb1*^−/−^ mice, *Bdkrb2*^−/−^ mice have elevated plasma BK, angiotensin II (AngII), and Ang-(1–7) (10, 11) (Figure 3). Also, plasma PK and plasminogen activator inhibitor-1 are increased, and factor XI is decreased (10). Antagonists to the Mas receptor (A-779) or AT2R (PD123319) are able to correct the delay in thrombosis occlusion to normal and both antagonists together are not additive (11). Furthermore, unlike *Klkb1*^−/−^ mice, *Bdkrb2*^−/−^ mice have long bleeding times (10, 11). Their plasma 6-keto-PGF1α levels (259 ± 42 pg/ml) are significantly higher than *Klkb1*^−/−^ mice (129 ± 12 pg/ml) (*p* < 0.003) and normals (75 ± 10 pg/ml) (7, 11). Twofold higher 6-keto-PGF1α levels produce the long bleeding times and the selective platelet function defect in *Bdkrb2*^−/−^ mice (11). *Bdkrb2*^−/−^ mice have a selective GPVI activation defect to collagen-rich peptide and convulxin. They manifest reduced murine activated integrin expression using the JON/A antibody and P-selectin expression. They also have a spreading defect on collagen, fibrinogen, and GFOGER, a β1 integrin adhesive peptide (11). On the other hand, *Bdkrb2*^−/−^ mice have normal thrombin- and ADP-induced platelet activation and fibrinogen binding, respectively. Finally, the cyclooxygenase inhibitor, nimesulide, shortens the bleeding time to normal and corrects the time to thrombosis in these animals. However, the platelet inhibition mechanism in *Bdkrb2*^−/−^ mice is not complete story for their delayed thrombosis times.

*Bdkrb2*^−/−^ mice also have elevated Sirt1 and KLF4 mRNA in their vessel wall. We suspect that these animals too have reduced TF in their vessel walls. In support of that assessment, a recent investigation shows that BK reduces vessel wall TF *via* cell activation and this translates into reduced thrombosis risk (13). A summary of this mechanism for thrombosis delay in *Bdkrb2*^−/−^ mice is shown in Figure 3.

## Summary

**Figure 4:**
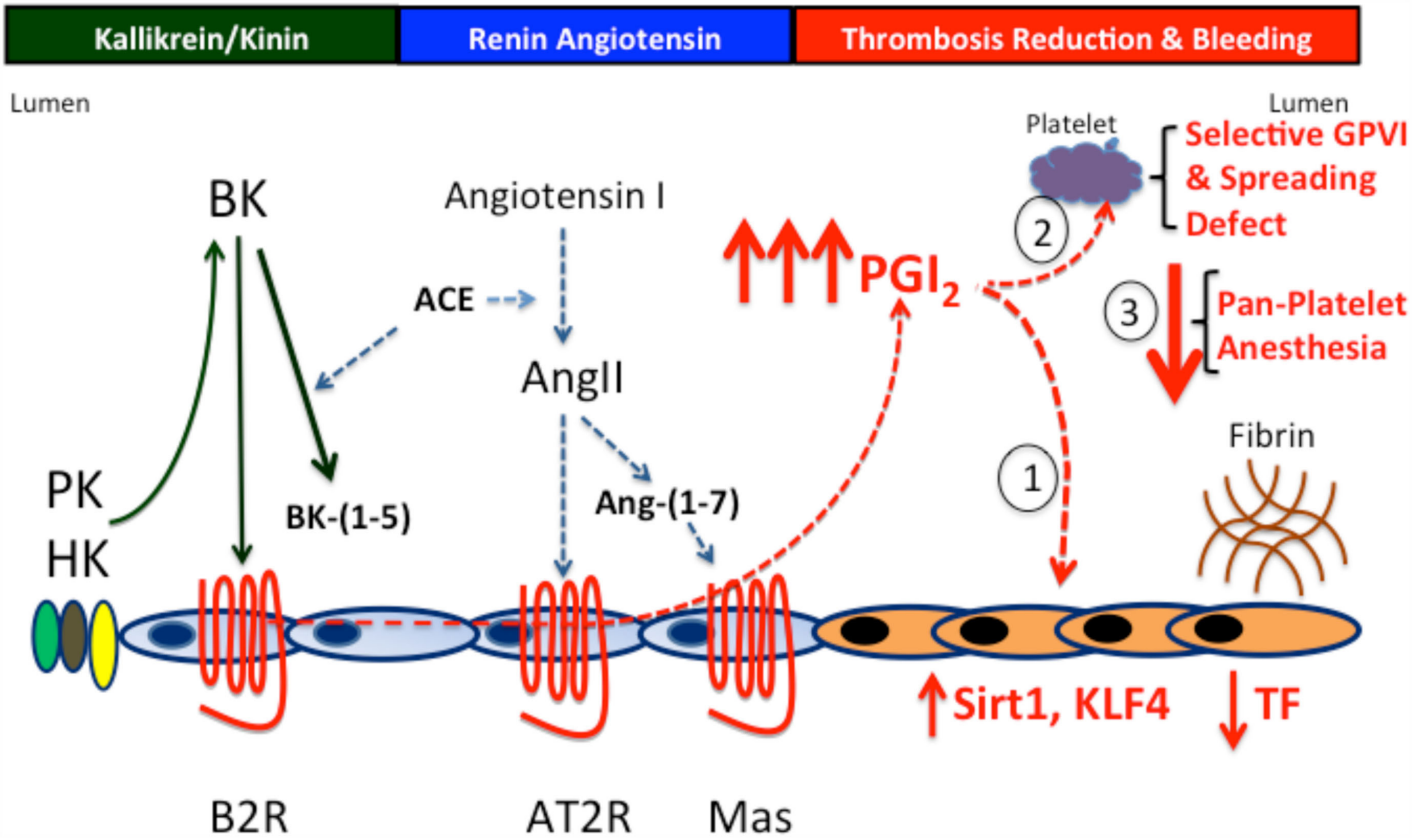
**The prostacyclin axis-induced thrombosis protection**. PK and B2R deficiency produces thrombosis protection through the prostacyclin axis. In the absence of PK or the B2R, there is increased prostacyclin production due to overexpression of the AT2R and/or Mas receptors to compensate for reduced or absent B2R. Prostacyclin induces a graded increase in thrombosis protection. First, at levels up to twofold increased, it effects the vessel wall reducing TF production. Second, at levels up to twofold to threefold increased, it downregulates vessel wall TF and induces a selective platelet function defect of reduced GPVI activation and spreading on collagen- and integrin-binding adhesive glycoproteins. Finally, at levels greater that threefold, prostacyclin produces the overall platelet anesthesia generally recognized with it.

In conclusion, we have uncovered in our detailed mechanistic studies on the *Klkb1*^−/−^ and *Bdkrb2*^−/−^ mice, a previously unappreciated thrombo-protective mechanism. BK through the B2R receptor, AngII through the AT2R, or Ang-(1–7) through Mas receptor has the ability to elevate prostacyclin in a graded fashion. Graded elevation of prostacyclin has a graded increase in thrombo protection. First, it stimulates expression of vasculoprotective transcription factors Sirt1 and KLF4 to downregulate vessel wall TF when increased 1.5- to 2-fold (Figure 4). Second, higher elevations (twofold to threefold increases) result in a selective platelet GPVI activation and spreading defect (Figure 4). These higher levels of prostacyclin lengthen bleeding times. Finally, higher concentrations of prostacyclin provide a general platelet anesthesia and give increased risk to bleed. Modulating vessel wall TF only through these three GPCRs may provide a novel approach to reduce thrombosis risk without enhanced risk to bleed.

## Author Contributions

Dr. AS wrote the manuscript and is fully responsible for it.

## Conflict of Interest Statement

The author declares that the research was conducted in the absence of any commercial or financial relationships that could be construed as a potential conflict of interest.
